# New records of sharks (Elasmobranchii) from the Andaman and Nicobar Archipelago in India with notes on current checklists

**DOI:** 10.3897/BDJ.6.e28593

**Published:** 2018-09-10

**Authors:** Zoya Tyabji, Rima W. Jabado, Dipani Sutaria

**Affiliations:** 1 Andaman Nicobar Environment Team, Port Blair, India Andaman Nicobar Environment Team Port Blair India; 2 Centre for Wildlife Studies, Bengaluru, India Centre for Wildlife Studies Bengaluru India; 3 Gulf Elasmo Project, Dubai, United Arab Emirates Gulf Elasmo Project Dubai United Arab Emirates; 4 James Cook University, Queensland, Australia James Cook University Queensland Australia

**Keywords:** biodiversity, elasmobranchs, range extensions, fishery-dependent survey, review

## Abstract

The diversity of sharks occurring off the Andaman and Nicobar Archipelago in India has received increased attention in recent years. Yet, available checklists are out of date, often with inaccurate information and a number of commercially important species have not been documented through research and fish landing surveys. Here we report on shark species examined during fish landing surveys conducted from January 2017 to April 2018. Records of twelve previously unreported species from the archipelago are presented and include the bignose shark (*Carcharhinus altimus*), pigeye shark (*Carcharhinus amboinensis*), bull shark (*Carcharhinus leucas*), snaggletooth shark (*Hemipristis elongata*), slender weasel shark (*Paragaleus randalli*), Arabian smoothhound shark (*Mustelus mosis*), Indonesian houndshark (*Hemitriakis indroyonoi*), sand tiger shark (*Carcharias taurus*), Indonesian bambooshark (*Chiloscyllium hasseltii*), tawny nurse shark (*Nebrius ferrugineus*), dwarf gulper shark (*Centrophorus atromarginatus*), and the Indonesian shortsnout spurdog (*Squalus hemipinnis*). These records increase the reported shark species for the archipelago from 47 to 59 and for India from 114 to 116. Additionally, a size extension in the total length of *C. hasseltii* by 27 cm and of *P. randalli* by 8 cm is reported. Owing to the bio-geographical location of these islands, species diversity around the archipelago is unique and appears to overlap with that of southeast Asia. With increasing reports of over-exploitation and the operation of a targeted shark fishery by distant water fleets in these waters, the limited information on shark diversity from this region is of concern. Systematic and long-term monitoring of catches, combined with accurate species identification, is crucial to provide information on management measures.

## Introduction

The waters of India harbour an estimated 114 shark species (Akhilesh et al. 2014, Sutaria et al. 2015) from more than 500 globally known species (Weigmann 2016), of which 47 have been reported from the Andaman and Nicobar Archipelago (hereafter referred to as ‘the archipelago’) (Table 1, Rajan et al. 2012, Varghese et al. 2015, Rajan et al. 2016, Pradeep et al. 2017a, Pradeep et al. 2017b). Seen as a fishery resource to be exploited, much shark research effort in mainland India has focused on catch effort and landing volumes (Akhilesh et al. 2014, Bineesh et al. 2016). Around the archipelago, large gaps remain in our understanding of shark resources with little research focused on species diversity across biogeographic zones, biology, stock structure and their socio-economic value (Rajan et al. 2016). Such information, combined with accurate species identification, is crucial in order to provide information on and support future management measures (Elphick 2008, Simpfendorfer et al. 2011).

Oceanic islands are highly productive, harbour high species diversities and may function as critical stops on the ontogenetic or annual migratory route of species, serving as important breeding or feeding grounds (Ashmole and Ashmole 1967, Carr et al. 1974, Das and Afonso 2017, Engel and Martin 2009, Olavarría et al. 2007). The archipelago is comprised of true oceanic islands that lie on the zone of tectonic contact between the Indian and eastern Asian plates (Mohanraj et al. 2010, Ripley and Beehler 1989). Lying closer to Southeast Asia than to peninsular India, the archipelago presents an ecological niche for species overlapping between these two regions (Ashraf 2006, Ripley and Beehler 1989, Mohanraj et al. 2010). Indeed, the Andaman Sea is believed to have a high diversity and unique faunal composition of fish and shark species and has been designated as a priority area for shark conservation (Lucifora et al. 2011, Satapoomin 2011).

Due to the distance of the archipelago from peninsular India, it has received limited attention in terms of ecological monitoring of its fisheries resources. Prior to the 1940s, there was no organised fishing sector on the archipelago (Ganapathiraju 2012). The indigenous tribes lived in hunter-gatherer societies and subsistence fishing formed a small component of these activities (Hornby et al. 2015, Kumaran 1973). To fully utilise marine resources and develop the fisheries sector, the Directorate of Fisheries introduced a ‘Fishermen Settlement Scheme’ in 1955 and settled fisher families on the Andaman Islands (Advani et al. 2013). Fishing for sharks started in the 1960s, in parallel with the initiation of targeted shark fishing on mainland India (James 1973). However, due to limited local demand for sharks on the archipelago, there was no impetus to develop a targeted shark fishery (James 1973). In the 1970s, only a few fishermen targeted sharks and were predominantly from the *Telegu* community from Andhra Pradesh on the east coast of mainland India (Advani et al. 2013). From the 1980s onwards, as consumption of shark meat and the fin trade industry developed on mainland India, shark fisheries on the archipelago developed to supply the export market. Fishermen were reported to fin sharks due to the rise in demand for their fins and the limited local demand for shark meat (Vivekanandan 2001), while deepsea sharks were increasingly targeted and retained for their liver oil (Akhilesh et al. 2011). Presently, in addition to sharks being caught as incidental catch in multi-gear fisheries, the archipelago still has one of the few targeted shark fisheries in Indian waters (Rajan et al. 2012). Furthermore, incursions from fishermen, originating from Andhra Pradesh and Tamil Nadu in India, Sri Lanka, Taiwan and Indonesia, are frequent and likely impacting local stocks (Advani et al. 2013, Ganapathiraju 2012). While the fisheries sector continues to expand, the implementation of existing fisheries regulations is minimal (Advani et al. 2013) and indications that shark stocks are impacted by these fisheries are rising, yet there is limited monitoring of shark landings.

Reporting by the Andaman and Nicobar Islands Directorate of Fisheries has broadly focused on commercial fish stocks and does not include species-specific or even group-level categories for chondrichthyans and all shark, ray and chimaera landing volumes are lumped together (Advani et al. 2013). This data limitation does not allow for the assessment of the species composition of landings or mortality levels, creating a gap in our understanding about the status of species. Furthermore, while some data have recently been collected on shark diversity on the archipelago (Rajan et al. 2012, Rajan et al. 2016, Varghese et al. 2015), there are still no systematic surveys of landings and much of the available literature is already out of date, often with inaccurate species identifications.

The main objectives of this study are to (1) update the species list of sharks occurring around the archipelago, (2) provide details of recent taxonomic revisions while correcting past misidentifications and (3) provide recommendations for future research and management opportunities to ensure the sustainability of shark stocks around these islands.

## Methods

**Study Area**

**Figure 1.**
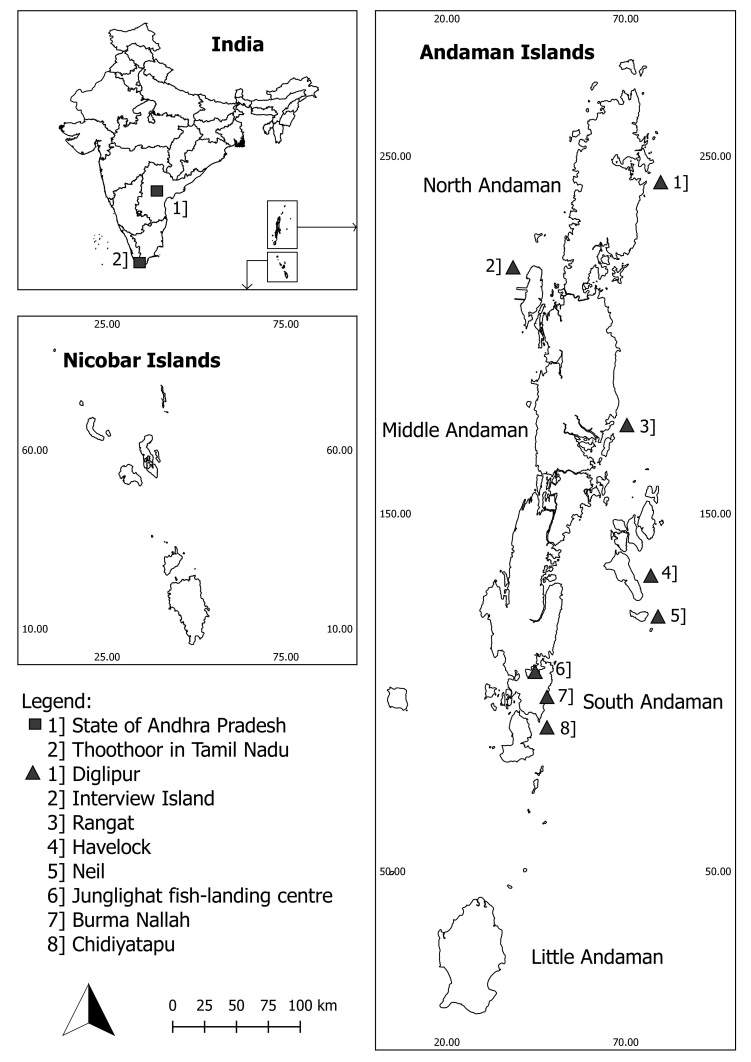
*Inset top left*: Map of India showing the location of the state of Andhra Pradesh, *Thoothoor* in Tamil Nadu and the Andaman and Nicobar Islands. *Inset bottom left*: Map of the Nicobar Islands. *Inset right*: Map of the Andaman Islands showing Junglighat and Burma Nallah, the two main fish-landing centres of South Andaman Islands and the fishing grounds.

The archipelago is considered unique in its geographical location and biogeography and is situated in the Indian Exclusive Economic Zone (EEZ) in the Bay of Bengal (Fig. 1, Rajan et al. 2012, Ripley and Beehler 1989). Geologically, it is part of a land mass of Southeast Asia lying closer to Myanmar and Indonesia, an area considered to be one of the marine biodiversity hotspots of the world (Roberts et al. 2002, Weigmann 2016). The archipelago comprises of the Andaman group (>325 islands, 24 inhabited, 6,408 km^2^) and the Nicobar group (21 islands, 13 inhabited, 1,841 km^2^), separated from each other by a ten-degree latitudinal channel and influenced by the south-western and north-eastern monsoons (May-December) (D’Souza et al. 2013). It accounts for 28% of India’s EEZ and 24% of India’s coastline, with its surrounding marine ecosystems shaping the entire political and social history of its inhabitants (Kar et al. 2011).

**Fish landing surveys**

Fish landing surveys were carried out at fish landing centres, namely, Junglighat situated in the capital city of Port Blair and Burma Nallah located south of Port Blair on the South Andaman Island (Fig. 1). Data collection was carried out on alternate days (weather permitting) from January 2017 to April 2018, with systematic surveys of the sites conducted during landings from 0600–1000 h and opportunistically from 1400–1600 h, respectively. During these surveys, vessels landing sharks were observed and landed sharks were sampled as long as time permitted (prior to fishermen and traders beginning processing of the catch).

Sharks were photo-documented to support identification using available literature (Compagno 1984,Compagno et al. 2005, Ebert et al. 2015, Jabado and Ebert 2015). Data were collected on sex, size, maturity stage and weight. Sex was determined by the presence or absence of claspers; maturity stage for males was noted depending on the size and condition of claspers (calcification levels); the presence of gravid individuals was noted by exposed pups or a bulge in the stomach; the presence of umbilical scars was noted; stretched total length (TL) (measured to the nearest centimetre using a measuring tape along the stretched body of the specimen) and weight (kg) (for small individuals using a hand-held circular weighing balance or when weights were provided by the fishermen using a circular weighing balance) were recorded whenever possible (Compagno 1984). Additionally, through informal discussions with the captains and crew members of the vessels that landed sharks, approximate fishing grounds were recorded for each catch.

**Literature review**

A comprehensive literature review was carried out by visiting the repositories of the Zoological Survey of India, Port Blair; Department of Fisheries, Andaman and Nicobar Islands; Fisheries Survey of India Port Blair; State Library of the Andaman; the Andaman Nicobar Environment Team (ANET); and through the Web of Science database. All available peer-reviewed articles and fisheries reports on shark diversity on the archipelago from 1871 to 2017 were collated and reviewed (Table 2). Species lists and available photographs within publications were verified by checking morphological features against descriptions and the updated nomenclature (Compagno 1984, Last et al. 2010, Ebert et al. 2015, Jabado and Ebert 2015, Weigmann 2016).

## Results

**Landing survey**

A total of 3864 sharks were recorded over 123 sampling days representing 36 species. Twelve species, previously unreported from the study area including Indonesian houndshark *Hemitriakis indroyonoi* and Indonesian shortsnout dogfish *Squalus hemipinnis*, two new records from the Indian EEZ, were recorded. Details of each of these twelve species are provided below with diagnostic characteristics that allow identification to the species level using Compagno et al. (2005), Ebert et al. (2015), Jabado and Ebert (2015).

1. CARCHARHINIFORMES - CARCHARHINIDAE - *Carcharhinus altimus* (Springer, 1950)

**Figure 2.**
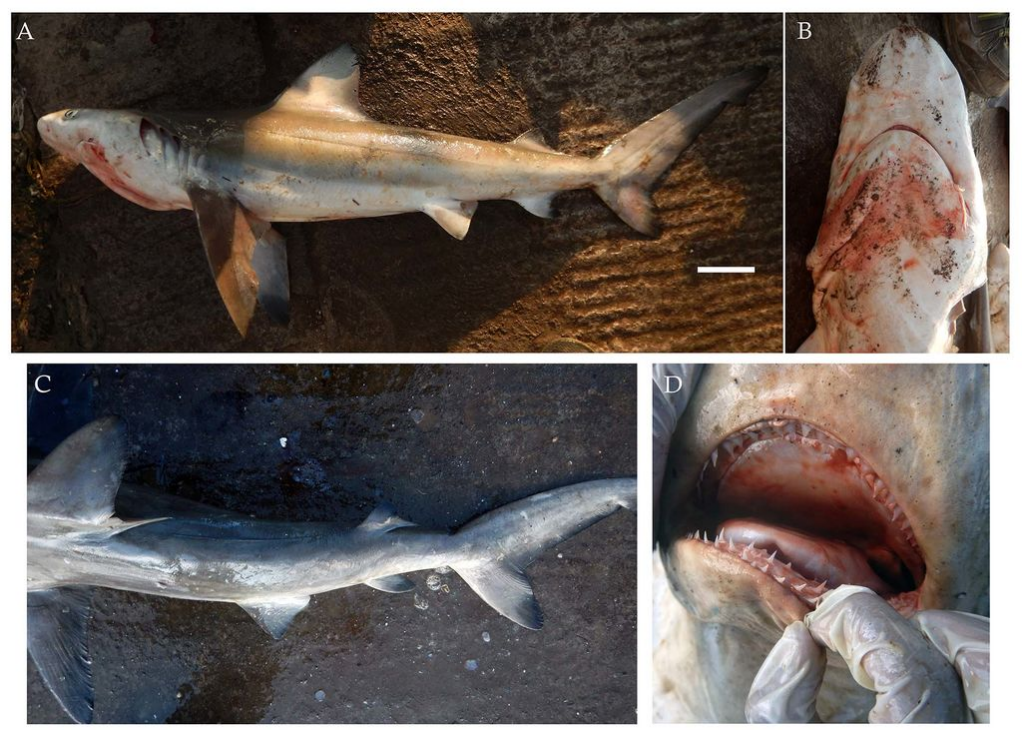
*Carcharhinus altimus.* **A.** Lateral view, scale bar = 100 mm, **B.** Underside of the snout **C.** Dorsal view showing the high interdorsal ridge **D.** Teeth.

From March 2017 to January 2018, three males and one female bignose shark *Carcharhinus altimus* (Fig. 2) were landed ranging in size from 90 cm to 237.5 cm TL with weights ranging from 2 kg to 93 kg. Two of the male specimens ranged in size from 103 cm to 128 cm TL and had the presence of an umbilical scar indicating they were recently born. The specimens were caught using longlines and gillnets from Neil and Havelock in South Andaman Islands at depths of approximately 20 m.

Diagnostic features: Large, broad, moderately rounded and long snout, equal to or greater than mouth width (Fig. 2B); first dorsal fin relatively tall, its origin over pectoral fin insertions or sometimes about half way along inner margins of pectoral fins (Fig. 2A); second dorsal fin high with short free rear tip, its origin slightly before anal fin origin; pectoral fins long and nearly straight; anal fin slightly larger than second dorsal fin; prominent high interdorsal ridge (Fig. 2C); moderately large and heavy cylindrical body (Fig. 2A).

2. CARCHARHINIFORMES - CARCHARHINIDAE - *Carcharhinus amboinensis* (Müller & Henle, 1839)

**Figure 3.**
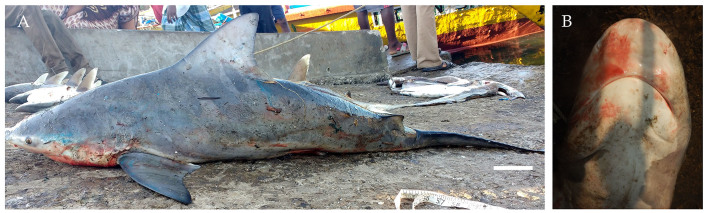
*Carcharhinus amboinensis* **A.** Lateral view, scale bar = 100 mm **B.** Underside of the snout.

From February 2017 to February 2018, thirteen male and nineteen female specimens of the pigeye shark, *Carcharhinus amboinensis* (Fig. 3) were landed, ranging in size between 134.5 cm to 295 cm TL with weights ranging from 12 kg to 210 kg. The specimens were caught at depths of 20-50 m using gill nets and longlines. They were fished from Diglipur and the Nicobar Islands, respectively.

Diagnostic features: Snout broad, short and bluntly rounded (Fig. 3B) with small eyes (Fig. 3A); mouth length less than mouth width (Fig. 3B); first dorsal fin high and triangular, height more than 3:1 times height of second dorsal fin (Fig. 3A); pectoral fins large and angular; interdorsal ridge absent; large, stocky and robust body (Fig. 3A).

3. CARCHARHINIFORMES - CARCHARHINIDAE - *Carcharhinus leucas* (Müller & Henle, 1839)

**Figure 4.**
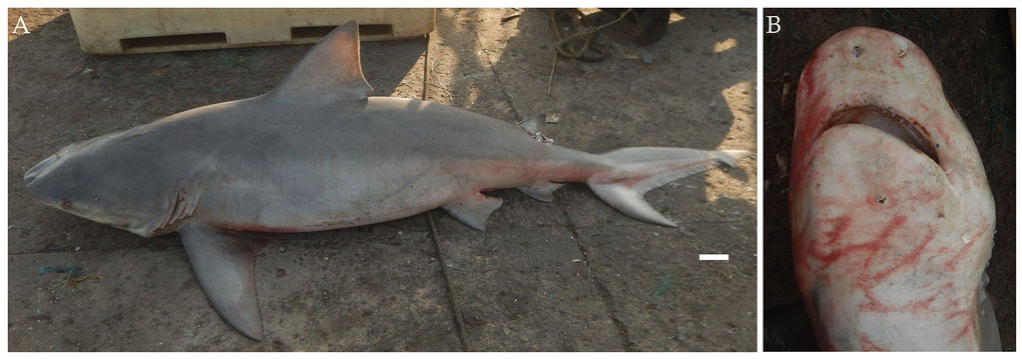
*Carcharhinus leucas* **A.** Lateral view, scale bar = 100 mm **B.** Underside of the snout.

From March 2017 to March 2018, ten male and fourteen female specimens of the bull shark, *Carcharhinus leucas* (Fig. 4) were landed ranging in size between 146 cm to 311 cm TL with weights ranging from 21 kg to 226 kg. The specimens were caught at depths of 20-50 m in trawl nets and longlines. They were fished from Interview Island, located to the West of North Andaman Islands and from the Nicobar Islands.

Diagnostic features: Snout broad, short and bluntly rounded (Fig. 4B); mouth length less than mouth width (Fig. 4B); first dorsal fin high and triangular, height equal or less than 3:1 times height of second dorsal fin (Fig. 4A); pectoral fins large and angular; interdorsal ridge absent; large, stocky and robust body (Fig. 4A).

4. CARCHARHINIFORMES - HEMIGALEIDAE - *Hemipristis elongata* (Klunzinger, 1871)

**Figure 5.**
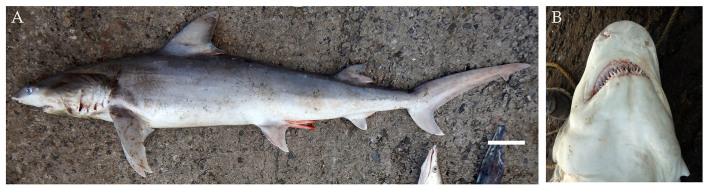
*Hemipristis elongata* **A.** Lateral view, scale bar = 100 mm **B.** Underside of the snout with protruding teeth.

From February 2017 to April 2018, eighteen male and ten female specimens of the snaggletooth shark, *Hemipristis elongata* (Fig. 5) were landed ranging in size from 93.1 cm to 211 cm TL with weights ranging from 4.4 kg to 53 kg. The specimens were fished from Diglipur in North Andamans, Rangat in Middle Andamans and Nicobar using hook and line and longlines.

Diagnostic features: Broadly rounded snout with protruding teeth when mouth closed (Fig. 5B); gill slits large and more than twice the length of eye length (Fig. 5A); all fins strongly curved (Fig. 5A); second dorsal fin about two thirds the size of first dorsal fin, its origin before smaller anal fin origin (Fig. 5A).

5. CARCHARHINIFORMES - HEMIGALEIDAE - *Paragaleus randalli* Compagno, Krupp & Carpenter, 1996

**Figure 6.**
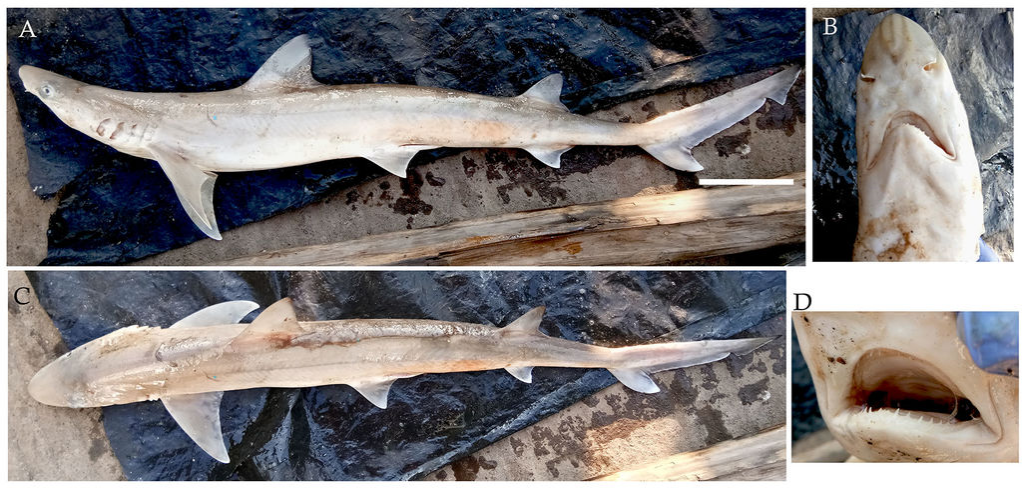
*Paragaleus randalli* scale bar = 100 mm **A.** Lateral view **B.** Snout showing a pair of lateral lines on rostrum **C.** Dorsal view **D.** Teeth.

From January 2017 to April 2018, one hundred and fifty three individuals of the slender weasel shark *Paragaleus randalli* (Fig. 6) were landed. Of these, 12 were gravid females ranging from 82.5 to 94 cm TL, with weights ranging from 2.5 kg to 3.7 kg; three were fully developed embryos ranging from 43.6 to 47.5 cm TL, one neonate measured 43.5 cm TL, all four with weights less than 0.5 kg and 80 mature specimens ranged from 68 to 95.8 cm TL with weights ranging from 0.5 kg to 4.05 kg. The specimens were caught using four different fishing gears - gillnets, hook and line, longlines and trawl nets. They were fished from Diglipur in North Andamans and Havelock in South Andamans, at depths of 15-20 m.

Diagnostic features: Snout with narrowly rounded tip and distinct dark lines (Fig. 6B); mouth long with long labial furrows and teeth visible when closed (Fig. 6B, C); large lateral eyes with nictitating eyelids; gill slit length equal to eye length; first dorsal fin origin slightly behind pectoral fin free rear tip origin (Fig. 6A); second dorsal fin size two-thirds the height of first dorsal fin, its origin over or slightly before anal fin origin (Fig. 6A); fins curved (Fig. 6A).

6. CARCHARHINIFORMES - TRIAKIDAE - *Mustelus mosis* Hemprich & Ehrenberg, 1899

**Figure 7.**
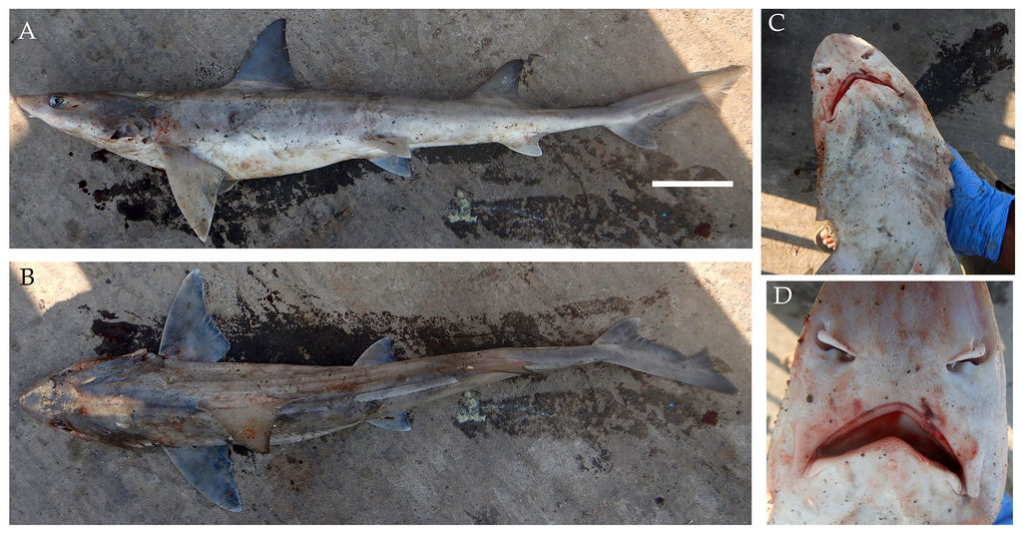
*Mustelus mosis* **A.** Lateral view, scale bar = 100 mm **B.** Snout showing unique mouth shape of the species **C.** Dorsal view **D.** Teeth.

On 4^th^ March 2017, three Arabian smoothhound sharks *Mustelus mosis* (Fig. 7) and on 23rd April 2018, four specimens were landed. All individuals were female ranging from 85.2 cm to 108.5 cm TL. They were captured by a mechanised dinghy using hook and line at a depth of 50 m. The hook size was a diameter of 6 cm and the bait used was the spotted sardine *Amblygaster sirm* (Walbaum), locally called *kappa tarni.*

Diagnostic features: Snout short and bluntly angular (Fig. 7B and C); long labial furrows with similar upper and lower lengths (Fig. 7C); teeth flattened and smooth (Fig. 7D); first dorsal fin origin behind pectoral fin insertion (Fig. 7A and B); second dorsal fin origin well behind pelvic fin rear tips but before anal fin (Fig. 7A and B); second dorsal fin with black tip (Fig. 7A).

7. CARCHARHINIFORMES - TRIAKIDAE - *Hemitriakis indroyonoi* W.T. White, Compagno & Dharmadi, 2009

**Figure 8.**
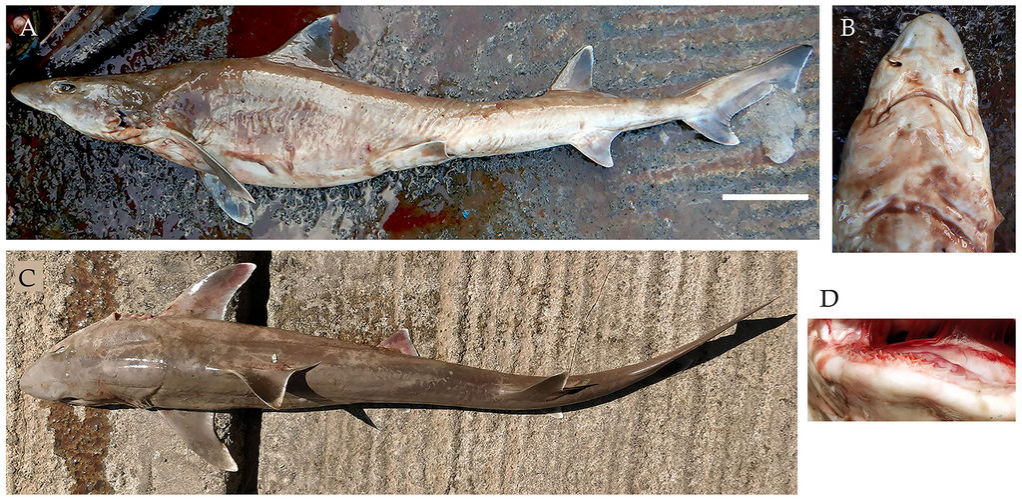
*Hemitriakis indroyonoi* **A.** Lateral view, scale bar = 100 mm **B.** Underside of snout **C.** Dorsal view **D.** Teeth of lower jaw.

In December 2017 and February 2018, two female Indonesian houndsharks were landed (Fig. 8). The specimens measured 100.6 cm and 105 cm TL and weighed 4.35 kg. They were caught using longline from Campbell Bay in Nicobar.

Diagnostic features: Snout long and narrow (Fig. 8B and C); rounded anterior nasal flaps, arched mouth (Fig. 8B); long upper labial furrows (Fig. 8B); falcate dorsal fins, pectoral fins semifalcate and anal fin strongly falcate (Fig. 8A and C); first dorsal fin origin over or behind pectoral fin rear tips; prominent white fin tips (Fig. 8A and C).

8. LAMNIFORMES – ODONTASPIDIDAE – *Carcharias taurus* Rafinesque, 1810

**Figure 9.**
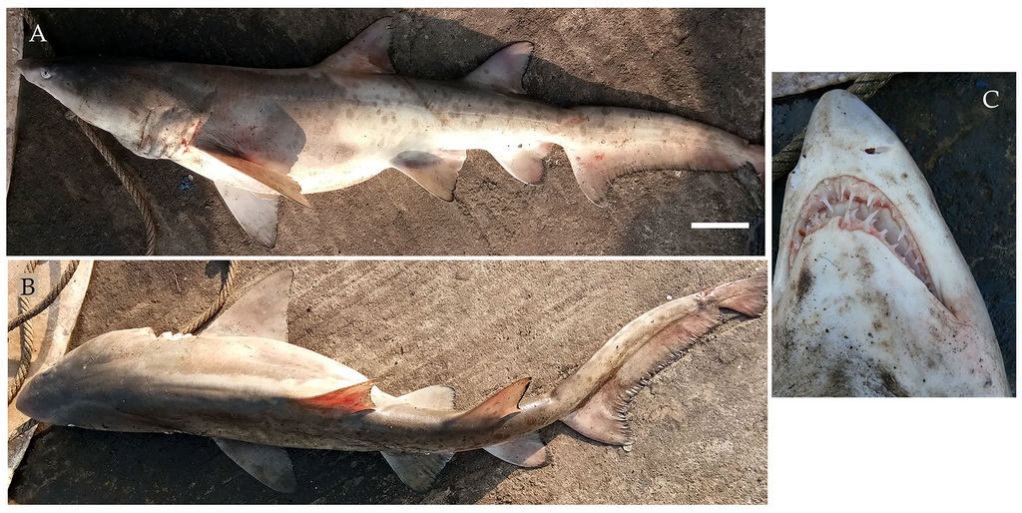
*Carcharias taurus* **A.** Lateral view, scale bar = 100 mm **B.** Dorsal view **C.** Snout with protruding teeth.

On 20^th^ March 2018, one female sandtiger shark *Carcharias taurus* (Fig. 9) was landed, measuring 129.4 cm TL. The specimen was caught in a gill net at depths of 20 m.

Diagnostic features: Conical short snout with large slender pointed teeth (Fig. 9C); small eyes and long mouth extending beyond eyes; first dorsal fin closer to pelvic fin than pectoral fin (Fig. 9A and B); large pelvic and anal fins similar in size (Fig. 9A); absence of interdorsal ridge (Fig. 9B); scattered darker spots on a large, heavy body (Fig. 9A).

9. ORECTOLOBIFORMES - HEMISCYLLIDAE - *Chiloscyllium hasseltii* Bleeker, 1852

**Figure 10.**
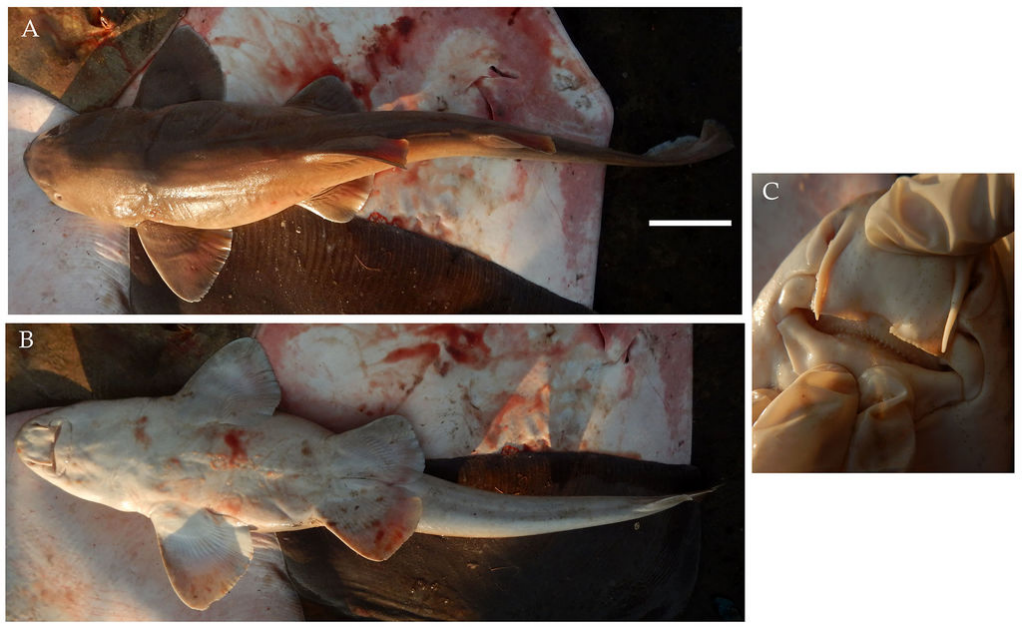
*Chiloscyllium hasseltii* **A.** Dorsal view, *s*cale bar = 100 mm **B.** Ventral view **C.** Mouth showing teeth and barbels.

On 20^th^ February, a female Indonesian bambooshark *Chiloscyllium hasseltii* (Fig. 10) was landed measuring 88 cm TL and weighing 3.2 kg. The specimen was caught by a trawl vessel fishing east from Havelock for six days in waters 12 nautical miles from shore at a maximum depth of 40 m.

Diagnostic features: Convex pectoral, pelvic and dorsal fins (Fig. 10A and B); long low anal fin set far back on long thick tail (Fig. 10A); origin of first dorsal fin over rear of pelvic fin base (Fig. 10A); unpatterned body with light edged fins (Fig. 10A).

10. ORECTOLOBIFORMES - GINGLYMOSTOMATIDAE - *Nebrius ferrugineus* (Lesson, 1831)

**Figure 11.**
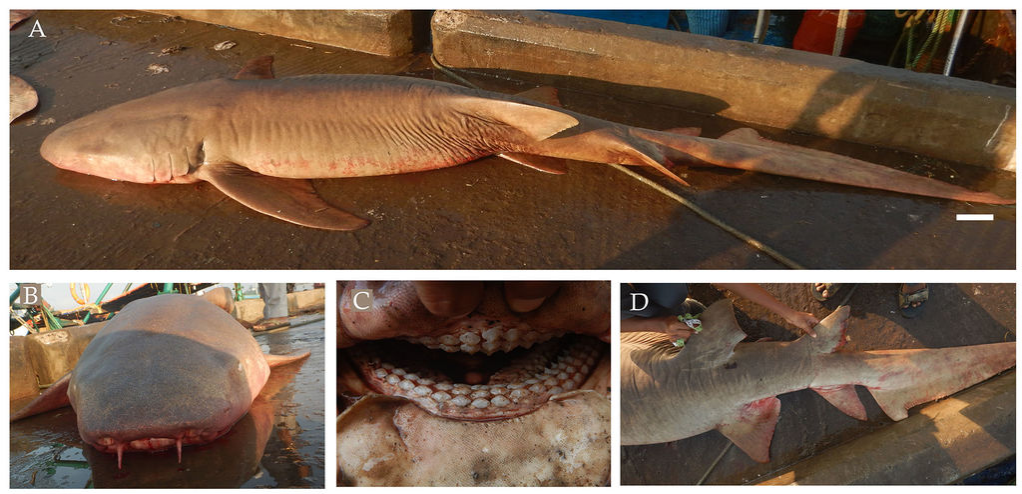
*Nebrius ferrugineus* **A.** Lateral view, scale bar = 100 mm **B.** Snout **C.** Teeth, **D.** Lateral view of the fins.

From February 2017 to March 2018, three male and two female specimens of tawny nurse shark *Nebrius ferrugineus* (Fig. 11) were landed, ranging in size from 271 cm to 312.5 cm TL with weights ranging from 105 kg to 150 kg. The specimens were fished from *Chidiyatapu*, South Andaman, using gill nets and trawl nets at depths of 20 m to 50 m.

Diagnostic features: Rounded snout with transverse, subterminal mouth well in front of eyes (Fig. 11A and B); small eyes with spiracles smaller than eyes; angular dorsal fin set back on the body (Fig. 11A); first dorsal fin slightly larger than second dorsal fin (Fig. 11A and D); anal fin origin behind second dorsal fin origin (Fig. 11A and D); caudal fin longer than a quarter of total length (Fig. 11A).

11. SQUALIFORMES - CENTROPHORIDAE – *Centrophorus atromarginatus* Garman, 1913

**Figure 12.**
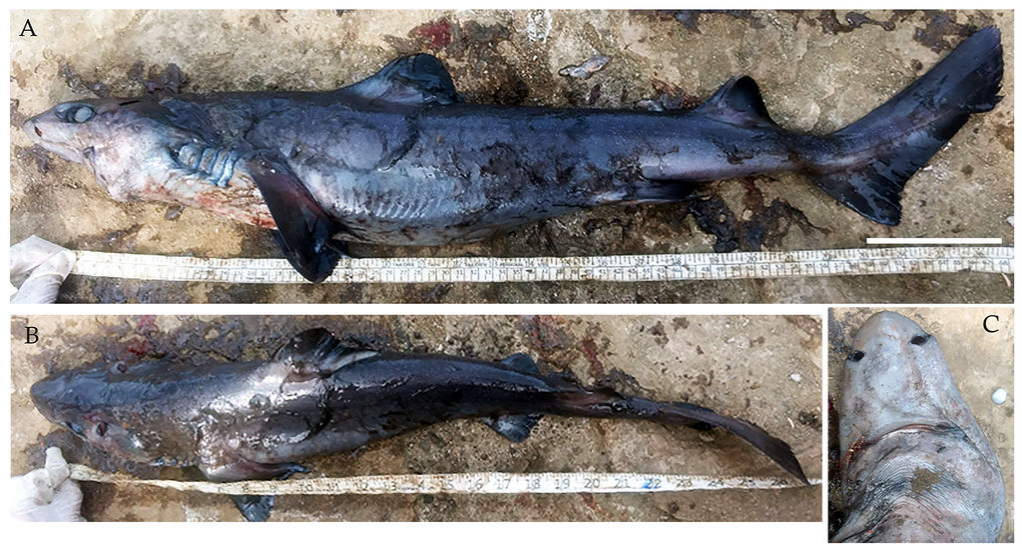
*Centrophorus atromarginatus* **A.** Lateral view, scale bar =100 mm **B.** Dorsal view **C.** Snout.

On 6^th^ September, a male dwarf gulper shark *Centrophorus atromarginatus* (Fig. 12) was landed. The specimen measured 72.5 cm TL and weighed 1.52 kg. It was caught by a longline gear targeting deep sea sharks at depths of more than 500 m at Diglipur.

Diagnostic features: Fairly long thick snout (Fig. 12C); rear tips of pectoral fins narrowly angular and greatly elongated (Fig. 12A and B); two dorsal fins with large groved spines (Fig. 12A); spine base of second dorsal fin over pelvic fin inner margins of rear tips (Fig. 12A); smooth skin with prominent blackish markings on all fins (Fig. 12A and B).

12. SQUALIFORMES - SQUALIDAE - *Squalus hemipinnis* White, Last & Yearsley, 2007

**Figure 13.**
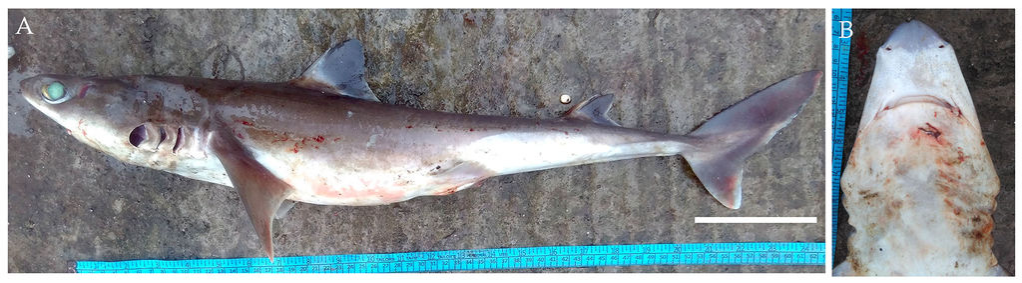
*Squalus hemipinnis* **A.** Lateral view, scale bar = 100 mm **B.** Snout.

On 21^st^ July, a female Indonesian shortsnout spurdog *Squalus hemipinnis* (Fig. 13) was landed. The specimen measured 66 cm TL and weighed 1.45 kg and was caught using hook and line.

Diagnostic features: Narrow, short, bluntly pointed snout (Fig. 13B); characteristic notch on second dorsal fin (Fig. 13A); sharply demarcated body colouration with slate grey above with dark area on head extending through to above gills (Fig. 13A); light-edged fins and caudal fin (Fig. 13A).

**Literature review**

We found 36 published accounts on sharks from the archipelago (Tables 1, 2). The earliest report of shark landings dates back to 1967 but species-specific information was not provided (James 1973). Since then, opportunistic or incidental reports of shark species in the fisheries have been reported via checklists and notes on new records (Table 2). However, there was no standard protocol followed or described for the above; and we found no systematic studies on diversity, ecology or the vulnerability of different shark species to local fisheries.

In addition, there have been frequent misidentifications and doubtful records of several shark species (Pillai and Parakal 2000, Sundararajan and Roy 2004, Rao 2009, Rajan et al. 2012, Rajan et al. 2016). Indeed, of the 47 shark species recorded from the archipelago (Table 1, Rajan et al. 2016), seven are unconfirmed or doubtful records for which there is no photographic evidence. These include the Ganges shark *Glyphis gangeticus*, smalleye hammerhead *Sphyrna tudes*, Pondicherry shark *Carcharhinus hemiodon*, blackspot shark *Carcharhinus sealei*, blue shark *Prionace glauca*, the shortnose spurdog *Squalus megalops* and the common thresher *Alopias vulpinus* (Sundararajan and Roy 2004, Rao 2009, Rajaram and Nedumaran 2009, Rajan et al. 2016). Additionally, when photographic evidence was available, sharks were found to be misidentified. For example, the slit-eye shark *Loxodon macrorhinus* is reported as the hardnose shark *Carcharhinus macloti* (Rajan 2003); the grey reef shark *Carcharhinus amblyrhynchos* is reported as the silvertip shark *Carcharhinus albimarginatus* (Rüppell, 1837) (Rajan 2003); the whitecheek shark *Carcharhinus dussumieri* as the blackspot shark *Carcharhinus sealei* (Rajan et al. 2012) and an unidentified weasel shark (Family Hemigaleidae) as the silky shark *Carcharhinus falciformis* (Rajan et al. 2012).

## Discussion

With systematic surveys carried out at fish landing sites, this study added twelve new species records to the known shark fauna of the Andaman and Nicobar Archipelago in a relatively short timeframe, highlighting the importance of monitoring landings at the species level. Ten of these species have been recorded and confirmed from mainland India (Akhilesh et al. 2014, Bineesh et al. 2016) and all have been confirmed from southeast Asia (Ali et al. 2013, Dharmadi et al. 2015, Howard et al. 2015, White et al. 2009, White et al. 2006). These first records of *S. hemipinnis* and *H. indroyonoi* increase the total species reported from Indian waters to 116. *Squalus hemipinnis* has been considered an endemic to Indonesia with the only available records from Bali, Java, Lombok and, possibly, Sumatra (White et al. 2007). Whereas, *H. indroyonoi* is a recently described species from Bali and Lombok in eastern Indonesia (White et al. 2009). Therefore, these distributional records, along with that of *C. hasseltii*, are species’ range extensions towards the Eastern Indian EEZ and highlight the overlap in species diversity of the archipelago with that of Southeast Asia.

The records of *C. hasseltii* and *P. randalli* increase their known total lengths from 61 cm to 88.5 cm TL and 83.6 cm to 95.8 cm TL, respectively (Compagno 2001, Goto 2005, Weigmann 2012). The record of 12 gravid *P. randalli* in February and March and two *C. altimus* neonates in early April also suggests that the waters around the archipelago are used as breeding and pupping grounds at least by these species. Indeed, the archipelago has a variable seafloor covering a wide range of depth gradients and harbours various marine habitats including mangroves and seagrass beds (D’Souza et al. 2013). Understanding the occurrence and distribution of shark species around the archipelago along with the use of these critical habitats as breeding or nursery grounds is crucial and warrants further research.

Accurate species identification is fundamental to monitoring ecological trends in populations, informing about and assessing conservation actions, designing and implementating management plans and evaluating the status of ecosystems and species (Austen et al. 2016, Beerkircher et al. 2009, Elphick 2008). Indeed, without accurate identification, it is not possible to produce species-specific accurate life history information or understand species richness, diversity and population trends, which are imperative for determining sustainable fishing levels and effectively managing populations (Cariani et al. 2017, Smart et al. 2016, White and Last 2012). While it is often difficult to identify a species in the field due to homoplasy, the phenotypic plasticity of morphological characters or even the presence of cryptic species (Dingerkus and DeFino 1983, White 2009, Cariani et al. 2017), the new records of sharks for this region had distinguishable features that could be visually confirmed and were supported by photo-documentation of key morphological characteristics (Fig. 2 to Fig. 13). In addition to the species listed here, some specimens, difficult to identify morphologically and requiring molecular analysis, are not reported here and this suggests that species diversity on the archipelago is much higher than 59. For some photographs that were unclear in past literature, such as *A. vulpinus* (Rajan 2003), it was not possible to confirm the species. While literature suggests that this species could occur in the Indian Ocean, its presence in the Bay of Bengal has not yet been confirmed. Publications that provide unvalidated information thus hinder our knowledge of shark species richness around the archipelago and make past literature doubtful and, to some extent, unusable. This is turn could result in wasting management resources and lead to erroneous conservation decisions. Surprisingly, the silky shark, *C. falciformis* listed as one of the most dominant bycatches in pelagic tuna longline fishery from the Andaman archipelago (Varghese et al. 2015) and also documented during this study, is absent from all earlier published checklists, suggesting that it has also likely been misidentified as another carcharhinid. The reported inaccuracies in the identification of species are not limited to sharks of the Andaman and Nicobar islands, as misidentifications of ray and guitarfish are also widespread in the published literature (e.g. Rajan et al. 2012). Moving forward, to ensure that literature focusing on the shark and ray fauna from the archipelago remains accurate, it is critical to, and a central recommendation of this paper, that correct methods of photo-documentation are used showing key morphological features to validate species identification (Compagno et al. 2005, Henderson and Reeves 2011, Jabado and Ebert 2015). Furthermore, we recommend integrating morphological identification with the use of molecular techniques (e.g. DNA barcoding; Ward et al. 2008, Jabado et al. 2015), at least for those species which are difficult to identify, to substantially reduce observer error (Bineesh et al. 2016).

Reported landings of elasmobranchs (sharks and rays) from the archipelago have quadrupled from 467 mt in 2001 to 2,124 mt in 2011 (Fisheries Survey of India 2012), with approximately 9 mt of shark fins and 467 mt of shark meat exported in 2011-2012, highlighting the importance of these fisheries and their contribution to the international shark fin trade (Director-Census Operations 2011). Owing to the expansion of fisheries on the archipelago, which quickly shifted from small-scale traditional and subsistence fisheries to an industrial and targeted fishery, the exploitation of many species, including sharks, has drastically increased (Advani et al. 2013). Species previously reported to be very common from the archipelago such as the sandbar shark *Carcharhinus plumbeus* and whitetip reef shark *Triaenodon obesus* (Advani et al. 2013) are now rarely recorded (Z. Tyabji unpubl. data) while other species, including the tiger shark *Galeocerdo cuvier*, have not been encountered in over 20 years (Andrews and Vaughan 2005, Advani et al. 2013).

Sharks are highly susceptible to fishing pressure and the lack of systematic monitoring of catch diversity and volumes, as well as the current lack of management, is a cause for concern with many species likely to have been overlooked and which could already have been overexploited (Stevens 2000, Ferretti et al. 2010, Dulvy et al. 2014). As this unmanaged exploitation continues, an increasing number of deepsea species are being landed from fisheries around the archipelago indicating that these are quickly expanding to offshore locations. Similar changes in fishing behaviour have led to the rapid collapse of deepsea shark stocks (*Centrophorus* spp.) along the west coast of India and the Maldives (Akhilesh et al. 2011, Jabado et al. 2018). In light of the potential impact these fisheries could be having on shark stocks around the archipelago and the current knowledge gap on species diversity, geographical distribution, ecology, life-history and species-specific landing volumes, we strongly recommend a precautionary approach to managing these resources.

## Figures and Tables

**Table 1.** Checklist of shark species occurring in the Andaman and Nicobar Islands.

	**Taxon**		**Common name**	**First report**	**Validity**
	**Family Alopiidae**				
1	*Alopias pelagicus* Nakamura, 1935		Pelagic Thresher	Rajan et al. 2012	Confirmed
2	*Alopias superciliosus* Lowe, 1841		Bigeye Thresher	Rajan et al. 2012	Confirmed
3	*Alopias vulpinus* (Bonnaterre, 1788)		Common Thresher	Rajan 2003	Needs confirmation
	**Family Carcharhinidae**				
4	*Carcharhinus albimarginatus* (Rüppell, 1837)		Silvertip Shark	Rajan 2003	Confirmed
5	*Carcharhinus altimus* (Springer, 1950)		Bignose Shark	This study	This study
6	*Carcharhinus amblyrhynchos* (Bleeker, 1856)		Grey Reef Shark	Talwar 1990	Confirmed
7	*Carcharhinus amboinensis* (Müller & Henle, 1839)		Pigeye Shark	This study	This study
8	*Carcharhinus brevipinna* (Müller & Henle, 1839)		Spinner Shark	Rao 2009	Confirmed
9	*Carcharhinus dussumieri* (Müller & Henle, 1839)		Whitecheek Shark	Herre 1941	Confirmed
10	*Carcharhinus falciformis* (Müller & Henle, 1839)		Silky shark	Varghese et al. 2015	Confirmed
11	*Carcharhinus hemiodon* (Müller & Henle, 1839)		Pondicherry Shark	Rajaram and Nedumaran 2009	Needs confirmation
12	*Carcharhinus leucas* (Müller & Henle, 1839)		Bull Shark	This study	This study
13	*Carcharhinus limbatus* (Müller & Henle, 1839)		Blacktip Shark	Rao 2004	Confirmed
14	*Carcharhinus longimanus* (Poey, 1861)		Oceanic Whitetip Shark	Rao 2004	Confirmed
15	*Carcharhinus macloti* (Müller & Henle, 1839)		Hardnose Shark	Talwar 1990	Confirmed
16	*Carcharhinus melanopterus* (Quoy & Gaimard, 1824)		Blacktip Reef Shark	Day 1871	Confirmed
17	*Carcharhinus plumbeus* (Nardo, 1827)		Sandbar Shark	Rajan et al. 2016	Confirmed
18	*Carcharhinus sealei* (Pietschmann, 1913)		Blackspot Shark	Talwar 1990	Needs confirmation
19	*Carcharhinus sorrah* (Müller & Henle, 1839)		Spottail Shark	Talwar 1990	Confirmed
20	*Galeocerdo cuvier* (Péron & Lesueur, 1822)		Tiger Shark	Rao 2004	Confirmed
21	*Glyphis gangeticus* (Müller & Henle, 1839)		Ganges Shark	Rao 2009	Needs confirmation
22	*Loxodon macrorhinus* Müller & Henle, 1839		Sliteye Shark	Talwar 1990	Confirmed
23	*Negaprion acutidens* (Rüppell, 1837)		Sharptooth Lemon Shark	Rao 2009	Confirmed
24	*Prionace glauca* (Linnaeus, 1758)		Blue Shark	Talwar 1990	Needs confirmation
25	*Rhizoprionodon acutus* (Rüppell, 1837)		Milk Shark	Day 1871	Confirmed
26	*Rhizoprionodon oligolinx* Springer, 1964		Grey Sharpnose Shark	Talwar 1990	Confirmed
27	*Scoliodon laticaudus* Müller & Henle, 1838		Spadenose Shark	Rao 2004	Confirmed
28	*Triaenodon obesus* (Rüppell, 1837)		Whitetip Reef Shark	Rao et al. 1997	Confirmed
	**Family Centrophoridae**				
29	*Centrophorus granulosus* (Bloch & Schneider, 1801)		Needle Dogfish	Sundararajan and Roy 2004	Confirmed
30	*Centrophorus atromarginatus* Garman, 1913		Dwarf Gulper Shark	This study	This study
31	*Centrophorus moluccensis* Bleeker, 1860		Smallfin Gulper Shark	Pradeep et al. 2017b	Confirmed
	**Family Ginglymostomatidae**				
32	*Nebrius ferrugineus* (Lesson, 1831)		Tawny Nurse Shark	This study	This study
	**Family Hemigaleidae**				
33	*Chaenogaleus macrostoma* (Bleeker, 1852)		Hooktooth Shark	Rao 2009	Confirmed
34	*Hemigaleus microstoma* Bleeker, 1852		Sicklefin Weasel Shark	Rajan et al. 2016	Confirmed
35	*Hemipristis elongata* (Klunzinger, 1871)		Snaggletooth Shark	This study	This study
36	*Paragaleus randalli* Compagno, Krupp & Carpenter, 1996		Slender Weasel Shark	This study	This study
	**Family Hemiscylliidae**				
37	*Chiloscyllium griseum* Müller & Henle, 1838		Grey Bambooshark	Rao 2004	Confirmed
38	*Chiloscyllium hasseltii* Bleeker, 1852		Indonesian Bambooshark	This study	This study
39	*Chiloscyllium indicum* (Gmelin, 1789)		Slender Bambooshark	Rao 2004	Confirmed
40	*Chiloscyllium punctatum* Müller & Henle, 1838		Brownbanded Bambooshark	Rajan et al. 1993	Confirmed
	**Family Lamnidae**				
41	*Isurus oxyrinchus* Rafinesque, 1810		Shortfin Mako	Rajan 2003	Confirmed
	**Family Odontaspididae**				
42	*Carcharias taurus* Rafinesque, 1810		Sandtiger shark	This study	This study
	**Family Proscyliidae**				
43	*Eridacnis radcliffei* Smith, 1913		Pygmy Ribbontail Catshark	Misra 1950	Confirmed
44	*Proscyllium magnificum* Last & Vongpanich, 2004		Magnificent Catshark	Kumar et al. 2015	Confirmed
	**Family Pseudocarchariidae**				
45	*Pseudocarcharias kamoharai* (Matsubara, 1936)		Crocodile shark	Pradeep et al. 2017b	Confirmed
	**Family Rhincodontidae**				
46	*Rhincodon typus* Smith, 1828		Whale Shark	Rajan et al. 2016	Confirmed
	**Family Scyliorhinidae**				
47	*Apristurus investigatoris* (Misra, 1962)		Broadnose Catshark	Misra 1962	Confirmed
48	*Bythaelurus hispidus* (Alcock, 1891)		Bristly Catshark	Alcock 1891	Confirmed
49	*Cephaloscyllium silasi* (Talwar, 1974)		Indian Swellshark	Kumar et al. 2016	Confirmed
	**Family Sphyrnidae**				
50	*Eusphyra blochii* (Cuvier, 1816)		Winghead Shark	Day 1871	Confirmed
51	*Sphyrna lewini* (Griffith & Smith, 1834)		Scalloped Hammerhead	Rajan 2003	Confirmed
52	*Sphyrna mokarran* (Rüppell, 1837)		Great Hammerhead	Rao 2004	Confirmed
53	*Sphyrna tudes* (Valenciennes, 1822)		Smalleye Hammerhead	Rao 2009	Needs confirmation
54	*Sphyrna zygaena* (Linnaeus, 1758)		Smooth Hammerhead	Devi and Rao 2003	Confirmed
	**Family Stegostomatidae**				
55	*Stegostoma fasciatum* (Hermann, 1783)		Zebra Shark	Rao et al. 2000	Confirmed
	**Family Squalidae**				
56	*Squalus hemipinnis* White, Last & Yearsley, 2007		Indonesian Shortnose Spurdog	This study	This study
57	*Squalus megalops* (Macleay, 1881)		Shortnose Spurdog	Sundararajan and Roy 2004	Needs confirmation
	**Family Triakidae**				
58	*Hemitriakis indroyonoi* W.T. White, Compagno & Dharmadi, 2009		Indonesian Houndshark	This study	This study
59	*Mustelus mosis* Hemprich & Ehrenberg, 1899		Arabian Smoothhound Shark	This study	This study

**Table 2.** Literature published on the diversity of sharks in the Andaman and Nicobar Islands, India.

**Authors**	**Title**	**Journal**	**Remarks**
Day 1871	On the fishes of the Andaman Islands	Zoological Society of London	Survey
Alcock 1891	Pisces: Natural history notes from H.M. Indian marine survey steamer 'Investigator'	The Annals and Magazine of natural history	Survey
Herre 1941	List of the fishes known from the Andaman Islands	Memoirs of the Indian Museum	Checklist
Misra 1950	New species of scyliorhinid from Andaman sea	Zoological Survey of India	New record
Misra 1962	A new scyliorhinid fish from the collections of the R.I.M.S. Investigator	Proceedings of the All-India Congress of Zoology	New record
James 1973	Living resources of the seas around India	Central Marine Fisheries Institute	Fisheries - opportunistic
Talwar 1990	Fishes of the Andaman and Nicobar Islands	Journal of Andaman Science Association	Checklist
Sivasubramaniam 1992	Pelagic sharks in the Indian Ocean	Bay of Bengal news	Fisheries - opportunistic
Rajan et al. 1993	New records of rare fishes from Andaman Islands	Journal of Andaman Science Association	New record
Hanfee 1996	The trade in sharks and shark products in India - a preliminary survey	TRAFFIC report	Fisheries - opportunistic
Rao et al. 1997	New records of fishes from the Andaman and Nicobar Islands	Environmental Ecology	New record
John and Somvanshi 2000	Atlas of tunas, bill fishes and sharks in the Indian Exclusive Economic Zone around ANI	Fisheries Survey of India	Checklist
Rao et al. 2000	An account of ichthyofauna of Andaman and Nicobar Islands	Zoological Survey of India	Checklist
Raje et al. 2002	Elasmobranch fisheries of India - an appraisal	Central Marine Fisheries Institute	Fisheries - opportunistic
Rao 2004	Guide to reef fishes of Andaman and Nicobar Islands	Zoological Survey of India	Guide
Devi and Rao 2003	Poisonous and venomous fishes of Andaman Islands	Zoological Survey of India	Identification guide
Venkataraman et al. 2003	Handbook on sharks of Indian waters	Zoological Survey of India	Checklist
Rajan 2003	A field guide to marine food fishes of Andaman and Nicobar Islands	Zoological Survey of India	Fisheries - opportunistic
Sundararajan and Roy 2004	Distributional records and biological notes on two deep sea shark from Andaman waters	Journal of Andaman Science Association	Fisheries - opportunistic
John and Varghese 2009	Decline in CPUE of Oceanic Sharks in the Indian EEZ	Proceedings to the Indian Ocean Tuna Commission	Fisheries - opportunistic
Rao 2009	Checklist of fishes of Andaman and Nicobar Islands	Environmental Ecology	Checklist
Rajaram and Nedumaran 2009	Ichthyofaunal diversity in Great Nicobar Biosphere Reserve	Journal of Threatened Taxa	Checklist
Sinha et al. 2010	Spatio-temporal distribution, abundance and diversity of oceanic sharks occurring in the ANI	Zoological Survey of India	Fisheries - survey
Kar et al. 2011	Bycatch in tuna longline fishery in the Indian EEZ around Andaman and Nicobar Islands	Proceedings to the Indian Ocean Tuna Commission	Fisheries - opportunistic
Rajan et al. 2012	Diversity and abundance of Chondrichthyes in the Andaman and Nicobar Islands	Ecology of faunal communities on the Andaman and Nicobar Islands	Checklist
Sajeevan and Sanadi 2012	Diversity, distribution and abundance of oceanic resources around Andaman and Nicobar Islands	Indian Journal of Fisheries	Fisheries - opportunistic
Advani et al. 2013	Emergence and transformation of marine fisheries in the Andaman Islands	Dakshin Foundation and ANET	Fisheries - literature review
Rajan et al. 2013	Fishes of Andaman and Nicobar Islands: A checklist	Journal of Andaman Science Association	Checklist
Akhilesh et al. 2014	Checklist of Condrichthyes in Indian waters	Journal of Marine Biological Association of India	Checklist
Varghese et al. 2015	Diversity, abundance and size structure of pelagic sharks caught in tuna longline survey in the Indian seas	Indian Journal of Geo-Marine Science	Survey
Kumar et al. 2015	First report of Magnificent catshark, *Proscyllium magnificum* Last and Vongpanich, 2004, from Bay of Bengal, Indian EEZ	World Journal of Fish and Marine Sciences	New record
Bineesh et al. 2016	DNA barcoding reveals species composition of sharks and rays in the Indian commercial fisheries	Mitochondrial DNA	Checklist
Rajan et al. 2016	First incidence of three sharks off Andaman Islands, India	Journal of Andaman Science Association	New record
Kumar et al. 2016	New biogeographic data and DNA barcodes for the Indian swellshark, *Cephaloscyllium silasi* (Talwar, 1974) from Andaman waters	Acta Ichthyologica Et Piscatoria	New record
Pradeep et al. 2017a	Report of the crocodile shark *Pseudocarcharias kamoharai* (Matsubara, 1936) from deep waters of the Andaman Sea	Marine Biodiversity	New record
Pradeep et al. 2017b	A first record of the Smallfin Gulper Shark *Centrophorus moluccensis* Bleeker, 1860 (Chondrichthyes: Squaliformes: Centrophoridae) from the Andaman and Nicobar waters, Indian EEZ	Journal of Threatened Taxa	New record
